# Prevalence of growth hormone receptor gene polymorphisms and their association with milk production and fertility-related traits of cross-bred dairy cows in Sri Lanka

**DOI:** 10.1080/10495398.2024.2307012

**Published:** 2024-02-02

**Authors:** Dameesha Indeewari Abeygunawardana, Ranasinghe Mudiyanselage Sajeewani Bimalka Kumari Ranasinghe, Sembukuttige Nimali Tharangani De Silva, Rathnayake Mudiyanselage Chandrasiri Deshapriya, Prathapasinghe Arachchige Gamika, Jayanthe Rajapakse

**Affiliations:** a Department of Basic Veterinary Sciences, Faculty of Veterinary Medicine and Animal Science, University of Peradeniya, Peradeniya, Sri Lanka; b Department of Livestock and Avian Sciences, Faculty of Livestock, Fisheries and Nutrition, Wayamba University of Sri Lanka, Makandura, Gonawila (NWP), Sri Lanka; c Department of Nano Science Technology, Faculty of Technology, Wayamba University of Sri Lanka, Kuliyapitiya, Sri Lanka; d Department of Animal Science, Faculty of Agriculture, University of Peradeniya, Peradeniya, Sri Lanka; e Department of Veterinary Pathobiology, Faculty of Veterinary Medicine and Animal Science, University of Peradeniya, Peradeniya, Sri Lanka

**Keywords:** Dairy cows, fertility, milk yield, *GHR*, SNP

## Abstract

This study investigated the association of selected growth hormone receptor (*GHR*) gene SNPs with selected fertility and milk production-related phenotypes of cross-bred dairy cows (*n* = 153) reared on three National Livestock Development Board farms in Sri Lanka. Selected cows were genetically screened for SNPs in the exon 08 (*n* = 153) and 5’ upstream (*n* = 118) regions of the *GHR* gene using the target sequencing method. The relationships between different genotypes and fertility traits (average calving interval, average number of services per conception, and age at first calving) and milk production-related traits (average total lactation yield, average lactation length, and average milk yield) were analyzed using the General Linear Model in SPSS. Among the identified Four *GHR* SNPs, rs1099014416 was significantly associated with average calving interval and age at first calving. Cows with GG genotype exhibited younger age at first calving (918.51 ± 113.42 days) and longer calving intervals (543.41 ± 43.29 days) compared to cows with GT (1275.18 ± 38.31, 515.09 ± 24.49 days) and TT (1212.89 ± 88.22, 364.52 ± 54.01 days) genotypes. Other SNPs did not show associations with the studied traits. SNP rs109014416 has the potential to be used as a genetic marker for fertility-related traits in the selection of cross-bred dairy cows in Sri Lanka which should be validated with a larger population.

## Introduction

Despite a significant expansion over the past few decades, the Sri Lankan dairy industry has not yet been able to become self-sufficient.^1^ Consequently, in order to meet the domestic demand, a substantial amount of foreign exchange must be spent each year to import powdered milk and other dairy products. To reduce this reliance on imports, the government has introduced and implemented several strategic actions to promote local milk production including the importation of high-yielding pregnant heifers of temperate origin for breeding and production purposes, genetic improvement of local herds, promoting private-sector investment through financial and technical assistance, establishing favorable farm gate prices for raw milk, etc.^2^ Some critical issues such as low productivity and feed shortage should have been resolved immediately but haven’t. This has hindered the success of those strategic actions.

One of the key persistent issues faced by the dairy sector in Sri Lanka is the low productivity of dairy animals. This is due to various reasons including a lack of systematic selection programme, improper breeding and poor nutrition.^2^ In Sri Lanka, livestock breeding programmes have been focused on the traditional phenotype-based selection method. But its accuracy is questionable and it could be one of the factors holding back the growth of the dairy industry. In comparison to the traditional, solely phenotype-based selection, the novel marker-assisted selection has several benefits.

Marker-assisted selection (MAS) uses molecular markers to select individuals with desirable genetic traits for breeding purposes.^3^ This approach has been successfully implemented in several developed countries and it is likely to be increasingly implemented in other countries aiming to achieve a rapid genetic gain in the productivity and sustainability of their dairy herds.^4^ In this method, the selection decisions are taken by assessing the genetic makeup of the animals therefore, its accuracy is much higher than the traditional phenotype-based selection strategy. Further, MAS enhances the response to selection even for the traits that have low heritability or are difficult to measure directly. To implement a MAS programme, it is necessary to identify molecular markers associated with the desired traits which can be used to screen breeding animals. Two commonly used methods for identifying molecular markers associated with desired traits are Genome-Wide Association Studies (GWAS) and the candidate gene approach.^5^

Among them, the candidate gene approach focuses on specific genes or pathways that are hypothesized to be involved in the trait of interest. This approach involves selecting a set of candidate genes based on prior knowledge or functional relevance and analyzing the genetic variation within those genes to identify associations with the trait of interest.^6^ The candidate gene approach is typically more hypothesis-driven than GWAS, and may be useful for identifying rare or highly penetrant genetic variants that are difficult to detect using GWAS.

The growth hormone receptor (GHR) plays a crucial role in regulating growth hormone signaling and has been shown to affect a variety of important physiological processes including growth, milk production, and reproduction. GH binds to GHR on target tissues to initiate its biological effects on lactogenesis, mammogenesis and metabolism of carbohydrates and lipids. The bovine GHR is encoded by a single gene located on chromosome 20^7^ and has been studied extensively as a potential candidate gene for milk production and fertility in cattle. Several studies have reported associations between genetic variants in the bovine *GHR* gene and milk production.^8^^,^^9^ In addition, the *GHR* gene has also been studied as a potential marker for fertility traits in dairy cattle. GH has been shown to play a role in regulating reproductive function, and genetic variations in the *GHR* gene have been linked to differences in reproductive performance in cattle.^10^

Therefore, this preliminary study aimed to investigate the prevalence of SNPs in selected fragments of the 5’ UTR and exon 08 regions of the *GHR* gene in crossbred dairy cows reared in the Northwestern Province of Sri Lanka. Additionally, the study aimed to determine the effects of those detected polymorphisms on the milk production and reproduction-related traits of these animals. Considering that the majority of dairy cows in Sri Lanka are crossbreeds,^11^ the results of this study will give useful information about the genetic diversity of the *GHR* gene in the local dairy cow populations and provide valuable insights into how these variations affect economically important traits. This knowledge could potentially be used to develop molecular marker panels for marker-assisted breeding programs, allowing for the accurate selection of cows with desirable genotypes to improve the productivity and profitability of the dairy industry in Sri Lanka to achieve expected goals.

## Materials and methods

### Experimental animals

A total of 153 cross-bred dairy cows reared in three National Livestock Development Board (NLDB) farms located in the Northwestern Province of Sri Lanka were examined in this study (Farm A −105 cows, Farm B −37 cows and Farm C −11 cows). Particularly, cross-bred multiparous cows with normal calving and no clinical reproductive abnormalities in the early postpartum period were selected. The number of cows selected from each farm was proportional to the total number of milking cows matching the above-mentioned selection criteria at the time of sample collection. In this study, any cross among indigenous cattle, temperate breeds (mainly Jersey), or improved Zebu breeds (mainly Sahiwal) was defined as cross-bred cows. All the animals were within their 2^nd^ to 6^th^ parity and their ages ranged between 3 and 12 years. All the farms adopted similar management practices under a semi-intensive management system. During the daytime cows freely grazed under coconut cultivation and they were confined into tie stall barns at night. Cows were supplemented with concentrates and cut grasses (CO-3 grass variety) depending on their milk yield. Accordingly, each liter of milk produced received 0.5 kg of commercial concentrate feed and 1 kg of chopped grasses. All cows had free access to drinking water. The cows were milked twice daily using either hand or machine milking methods. Hand milking was done by government-trained milkers.

Animals were mostly bred by artificial insemination using frozen semen of Jersey or Sahiwal bulls produced at Central Artificial Insemination Station, Kundasale, Kandy. After confirming the estrus and checking previous records, AI was done by trained field livestock officers using the recto-vaginal procedure in accordance with the AM-PM rule. The detection of estrus was done by livestock keepers by visual observation of behavioral signs and morphological changes. Around 40–45 days post insemination, the pregnancy was diagnosed by government veterinary surgeons by rectal palpation. When AI was not successful natural service was used.

### Blood sample collection and DNA extraction

The collection of blood samples and the subsequent extraction of DNA followed the methodology outlined in the study by Abeyunawardana et al. in 2023.^12^

### PCR amplification, DNA sequencing and genotyping

Two primer sets were developed to amplify the target regions of exon 08 and 5′ UTR of the *GHR* gene by using the Primer Express Software v2.0 (Applied Biosystems). Details of the targeted regions of the two genes, lengths of the amplified fragments and primer sequences used are described in Table A1.

The initial PCR amplification reaction was carried out in a total volume of 50 µL consisting of ddH_2_O, 1.5 mM of MgCl_2_, 200 µM of dNTPs, relevant forward and reverse primers (20 pmol),10 µl of 5X PCR buffer, 2 units of Taq DNA polymerase (Go taq, Promega, Madison, WI, USA) and around 1-2 µl of extracted DNA. The protocol started with an initial denaturing step of 95 °C for 3 min, followed by 35 cycles of denaturation at 95 °C for 30 sec, annealing at 57 °C for 30 sec, extension at 72 °C for 2 min and a final extension step at 72 °C for 5 min. The annealing temperatures and primer concentrations were then optimized separately for each gene fragment. Accordingly, for the 5′ UTR gene fragment, a primer concentration of 20 pmol and an annealing temperature of 61 °C and for the exon 08 gene fragment a primer concentration of 10 pmol and an annealing temperature of 58.6 °C were used. All the amplified PCR products were electrophoresed at 80 mV for 30 min in 0.8% agarose gels and visualized under ultraviolet transillumination with a 100 bp ladder running in parallel as a standard. Then they were purified and sequenced commercially (Macrogen Inc, Republic of Korea) for both directions. Mega 7 software and visual inspection of electropherograms obtained from Chromas software were used to detect SNPs.

### Data collection and trait definition

Details of each individual animal including breed, parity, dates of AIs or natural services, dates of calving, estimated monthly milk yield and days at milk for the respective month were recorded from their individual cow cards (history sheets) and milk production record books for up to 5 lactations. The recorded data were used to calculate three milk production-related traits (average milk yield, average lactation length and average total lactation milk yield) and three fertility traits (average calving interval, average number of services per conception, and age at first calving). The daily average milk yield for each cow was calculated for each lactation by dividing the sum of the total monthly milk yields by the total days in milk. The average milk yield per day for a single cow was the average of the above values of total lactations considered. This value was calculated using records from 2 to 6 lactations. For each lactation, the sum of total monthly milk yields was considered as the total lactation milk yield whilst the total number of days in milk was considered as the lactation length. Their average values were defined as the average total lactation milk yield and the average lactation length, respectively. The average value of the time between two consecutive calvings was defined as the average calving interval. The time between the first and second calvings was taken into consideration for the second parity cows. The average value of the total number of services (both natural and artificial inseminations) needed for each conception was defined as the average number of services per conception.

### Statistical analysis

The genotype frequencies for polymorphisms of each gene fragment were calculated by counting the individuals bearing respective genotypes and dividing them by the total number of genotyped individuals. Based on that, respective allele frequencies were calculated. Only 105 out of 118 total genotyped cows were included in the statistical analysis after excluding the cows with missing reproductive and milk production-related data. The associations between observed *GHR* genotypes and selected fertility and milk production-related traits were examined using the General Linear Model (GLM) of SPSS version 26.0 (SPSS Inc., Chicago, IL, USA). The genotypic differences were assessed by comparing the least significant differences. In here, genotype, farm and age category were considered as fixed factors. Parity was included as a covariate. The fitted final statistical model was as following:

$$Y_{\text{ijk}}=U+G_{i}+F_{j}+A_{k}+F_{j}xG_{i}+P_{l}+e_{\text{ijkl}}$$

Where *Y_ijk_* was the phenotypic value of traits (average milk yield, average lactation length, average total lactation milk yield, average calving interval, average number of services per conception and age at first calving), *U* was the population mean, *G_i_* was the fixed effect of genotypes, *F_j_* was fixed effect of the farm (farms A, B or C), *A_k_* was the fixed effect of age category (<4 years, 5-9 years, >9 years), *F_j_* x *G_i_* was the effect of farm genotype interaction, *P_k_* was the effect of the covariate (parity) and *e_ijk_* was a random residual error. For the age at first calving, the covariate, parity was removed from the model. For statistical analysis, SNPs with at least one genotype having less than two cows were not taken into consideration.

## Results

### Prevalence of SNPs in bovine GHR gene

A population of cross-bred dairy cows reared on three National Livestock Development Board farms was genetically screened for SNPs in the 5′ UTR (*n* = 118) and exon 08 regions (*n* = 153) of the growth hormone receptor gene using the target resequencing method. The presence of two previously known SNPs, rs109014416 and rs109231659 were initially tested in 118 crossbred dairy cows within the 211 bp fragment of the *GHR* 5’ UTR region. Sequencing results revealed the presence of both tested SNPs, rs109014416 and rs109231659 at genotypic frequencies of GG = 0.136, GT = 0.127, TT = 0.737 and GG = 0.788, GT = 0.136, TT = 0.076, respectively. Other than that, another SNP (rs134115846) was also observed in the cross-bred cows screened in the present study. The genotypic frequencies of SNP, rs134115846 were 0.356, 0.305 and 0.339 for AA, AG and GG genotypes, respectively. In the bovine *GHR* gene, a 354 bp fragment in the exon 08 region was sequenced to detect one previously known SNP, rs385640152. In the study population, the allele T was observed only in two animals, (one with heterozygous genotype, AT and one with homozygous genotype, AA) out of 153 tested animals. The reported SNPs and their genotype and allele frequencies are given in Table A2.

### Association between different genotypes of detected GHR gene SNPs and tested fertility traits

Table A3 summarizes the findings of the analysis performed to determine the relationship between genotypes of *GHR* gene SNPs and the assessed fertility traits. Genotypes of the SNP, rs109014416 detected in 5’ UTR region of *GHR* gene was found to be significantly associated with average calving interval (*P* = 0.041) and age at first calving (*P* = 0.014). In here, cows with GG genotype calved at a relatively younger age (918.51 ± 113.42 days) compared to the cows with TT and GT genotypes (1212.89 ± 88.22 and 1275.18  ± 38.31 days, respectively). Meanwhile, cows with GG genotype showed longer average calving intervals (534.41 ± 43.29 days) compared to GT and TT genotypes (364.52 ± 54.01 and 515.09 ± 24.49 days). There were no significant differences in average number of services per conception among the three genotypes related to SNP locus, rs109014416. However, the animals with heterozygous genotype GT had lower number of services per conception than the animals with GT and TT genotypes. No statistically significant difference was found among the genotypes related to the other detected SNPs, rs109231659 and rs134115846 for any of the fertility traits tested in this study.

### Association between different genotypes of detected GHR gene SNPs and tested milk production-related traits

No statistically significant difference among the genotype groups related to the *GHR* SNPs was observed for any of the milk production-related traits tested in this study (Table A4). Anyhow, the animals with the AA genotype for SNP, rs134115846, showed the highest average values for milk yield per day (6.20 ± 0.55 L/day), total lactation milk yield (1841.22 ± 244.54 L) and lactation length (284.51 ± 23.14 days), whereas the animals with the GG genotype showed the lowest average values for all of those traits. When the SNP rs109014416 was considered, animals with the TT genotype had longer lactation lengths (280.35 ± 15.39 days) and higher total lactation milk yields (1737.20 ± 168.76 L) than animals with the GG (1537.04 ± 324.62 L) and GT (1519.23 ± 380.39 L) genotypes. Meanwhile, animals with heterozygous genotype GT showed the lowest average values for all the tested milk production-related traits. For SNP, rs109231659 the highest average milk yield per day (8.12 ± 0.84 L/day) and the highest average total lactation yield (1894.00 ± 436.73) were observed in cows with TT genotype meanwhile the lowest values were observed in cows with GG genotype. The SNP detected in the *GHR* exon 08 region (rs385640152) was excluded from this analysis as it had genotype groups (AT and AA) with less than two members.

## Discussion

The long promotor region (5’ UTR) of the bovine *GHR* gene contains nine untranslated exons (1 A-1I) which can be alternately spliced into several GHR mRNA variants. As a result, it acts as a mediator in the regulation of *GHR* gene expression. Waters et al. (2011)^10^ reported this as a highly polymorphic gene region by discovering 31 putative SNPs in Holstein-Friesian cows. Among them, two SNPs, *GHR* 4.1 (rs109014416) and *GHR* 4.2 (rs109231659) were detected in a short 211 bp length fragment of the *GHR* 5’ UTR region of cows screened under the current study. In relation to rs109231659, the reference allele “G” was the most common allele in cross-bred dairy cows included in the study, meanwhile, the alternative allele “T” was the most common allele in Holstein-Friesian cows.^10^ In relation to rs109014416, the most prevalent allele in Holstein-Friesian cows was the reference allele “G”.^10^ Meanwhile, the alternative allele “T” was the most prevalent allele in cross-bred dairy cows of Sri Lanka.

The low frequency of the “A” allele at the F279Y (rs385440152) SNP locus of the *GHR* exon 08 region was common in most of the investigated dairy cattle populations [Holstein Friesian cows, F(A) = 0.13^10^, Jersey cows, F(A) = 0.15^13^, UK dairy cows F(A) = 0.20^14^ and Holstein–Friesian dairy cattle F(A) = 0.11^15^ including the screened population in this study [F(A) = 0.01]. When compared to other studies, the lowest frequency of the “A” allele at SNP locus rs385440152 was reported in the present study. Meanwhile, comparatively higher frequencies of allele “A” were reported in Germen Holstein cows, F(A) = 0.35^16^ and Chinese Holstein cattle F(A) = 0.36.^17^

Out of three SNPs detected in the *GHR* 5′ UTR region of cross-bred dairy cows of Sri Lanka, the effect of only one SNP (rs109014416) was significant for tested fertility traits (average calving interval and average age at first calving) but not for milk production-related traits (average milk yield, lactation length and total lactation milk yield). However, no statistically significant relationship was reported between the genotypes of this SNP with fertility and milk production traits in Holstein Friesian cows.^10^ Meanwhile, the effect of this SNP was significant for body condition score.^10^ The SNP, rs109231659 was not significantly associated with any of the traits tested in this study including average milk yield and total lactation milk yield. However, according to Waters et al. (2011)^10^ the most common allele, “T” at SNP locus rs109231659 was positively associated with lactation milk yield of Holstein-Friesian cows. As it was not associated with calving interval or functional survival, it has been highlighted as a potential marker for increasing milk yield without compromising reproductive performance. Simultaneously, the results of the current study support the positive association between the “T” allele and milk yield by revealing the highest average milk yield and total lactation milk yield in the TT genotype group (8.12 ± 0.84 L/day/cow and 1894.00 ± 436.73 L/lactation/cow), though it was not detected as a statistically significant effect.

These two SNPs are located close to each other (30 bp) but far from the reported untranslated exons of the 5′ UTR region. As a result, their function in transcription regulation cannot be predicted. However, these findings suggest the possibility of having more unreported untranslated exons in the 5′ UTR region that are important in the control of *GHR* gene expression.^10^ Furthermore, the polymorphic locus associated with these two SNPs has been identified as a probable binding site for certain transcription factors. The replacement of the reference alleles from the alternative alleles eliminates that binding sites and can affect the complex regulation of *GHR* gene expression.^10^ In here, the distance between the particular SNP locus and adjust promotors should also be considered. Moreover, the SNP locus rs109231659 has been identified as a major QTL for the somatic cell score of Italian Holstein Friesian heifers.^18^ Several association studies have also highlighted the importance of polymorphisms located in the *GHR* 5′ UTR region in the regulation of a wide range of economically important traits.^10^^,^^19^^,^^20^ Other than those two SNPs, rs134115846 was also found in the 5’ UTR region of bovine *GHR* gene. But it did not show any association with any of the traits tested in this study. Therefore, further investigations are required to evaluate the effect of those SNPs on other functionally important traits of cross-bred dairy cows of Sri Lanka.

*GHR* exon 08 SNP, rs385640152 was not considered for this association analysis due to having a minor allele frequency of 0.02. However, it has been extensively studied in several cattle populations and reported with a significant effect on milk yield related traits in Holstein dairy cows.^9^^,^^15^^,^^16^^,^^21^ According to Rahmatalla et al. (2011),^16^ the analysis of 1370 dairy cows confirmed a strong association of this SNP with milk yield, as well as fat, protein, and casein contents. Replacement of the neutral phenylalanine encoding “T” allele by the uncharged but polar tyrosine encoding “A” variant in the transmembrane domain of *GHR* led to a decrease in milk fat and protein yields as well as the somatic cell score.^16^ There is a hydroxyl group on the aromatic ring of tyrosine, which makes it less hydrophobic than neutral phenylalanine.^15^^,^^22^ This observation is also supported the findings of Rahmatalla et al.^16^ who reported higher 305 d milk fat, protein and lactose yields of AT genotype group than the AA genotype group of Chinese Holstein cattle. In contrast, studies by Blott et al.^21^, Rahmatalla et al.^16^ and Waters et al.^23^ provided significant evidence for the positive effect of the less common “A” allele on milk yield of Irish, German and New Zealand Holstein Friesian herds which have been extensively selected for greater milk production for several decades. Moreover, the locus related to *GHR* gene SNP, rs385440152 has been identified and validated as a strong QTL for different types of milk yield and composition-related traits. For example, this locus has been identified as a QTL for milk yield in Ayrshire cattle,^24^ Holstein and German Fleckvieh cattle,^25^ milk fat yield, fat percentage, protein percentage in German Fleckvieh and Holstein cattle.^25^ Furthermore, this polymorphism has been associated with a wide range of traits, including body composition and yield traits,^26^ feed intake, feed conversion, body energy traits,^14^ and calving interval,^23^ live weight in cattle^21^ and somatic cell score^16^ indicating the possibility of pleiotropic effect.

The effect of rs385640152 on milk yield and composition has been validated consistently across different cattle breeds and populations and it has been identified as a causative mutation which can be used as a molecular marker for dairy cattle selection programmes. Anyhow, the biological mechanisms behind the effects of this polymorphism on milk production have not been established yet and it is unlikely to be mediated by the JAK2-STAT5 pathway, the currently known major signaling pathway from the growth hormone receptor.

## Conclusions

The SNP, rs109014416 located at the 5’ UTR region of the *GHR* gene displayed a significant association with fertility traits, while no significant impact was observed on milk production traits. This suggests the potential utility of this SNP as a genetic marker for fertility-related traits in dairy cows. However, the reported association should be confirmed using a larger sample size.

## Data Availability

The datasets utilized and analyzed in this study are available upon reasonable request from the corresponding author.

## Appendices

**Table A1.** Primers used for PCR and sequencing of bovine *GHR* gene fragments.

Targeted region	Length of the amplified fragment	Primers (5’- 3’)
Exon 08	354 bp	F - AGGAAGTTTTATGTGGAACAGGAG
		R – ACCTGTGTTGTCTCTCTGTGG
5’ UTR	211 bp	F – TTGACCCTTGAACAAAATGGG
		R – CCTGACTCCTGCCTTCTTCAA

F: Forward; R: Reverse.

**Table A2.** Genotypic and allelic frequencies of SNPs in *GHR* gene of screened cross-bred dairy cattle population in Sri Lanka (*n* = 118 for *GHR* 5’ UTR fragment and *n* = 153 for exon 08 fragment).

Region	SNP	Presence in tested population	Nucleotide change	Genotypic frequencies (number of cows in each genotype)	Allelic frequencies	
					Major allele frequency	Minor allele frequency
5’ UTR	rs109014416	Yes	G→T	GG = 0.136 (16) GT = 0.127 (15) TT = 0.737 (87)	T = 0.801	G = 0.119
	rs109231659	Yes	G→T	GG = 0.788 (93) GT = 0.136 (16) TT = 0.076 (9)	G = 0.856	T = 0.114
	rs134115846	Yes	A→G	AA = 0.356 (42) AG = 0.305 (36) GG = 0.339 (40)	A = 0.508	G = 0.492
Exon 08	rs385640152	Yes	T→A	TT = 0.987(151) TA = 0.007 (1) AA = 0.007 (1)	T = 0.990	A = 0.010

**Table A3.** Association between different genotypes of detected SNPs in *GHR* gene 5’ UTR fragment and fertility traits.

SNP	Genotype	Services per conception	Age at first calving (days)	Average calving interval (days)
rs109014416	GG	1.61±0.25	918.51^a^±113.42	534.41^a^±43.29
	GT	1.17±0.31	1212.89^b^±88.22	364.52^b^±54.01
	TT	1.76±0.14	1275.18^b^±38.31	515.09^a^±24.49
rs109231659	GG	1.73±0.14	1264.09±40.62	484.76±21.72
	GT	1.45±0.28	1127.49±91.82	549.87±43.79
	TT	1.36±0.31	1121.11±103.20	362.64±49.42
rs134115846	AA	1.55±0.22	1255.80±55.51	470.67±39.15
	AG	1.70±0.23	1259.33±106.64	496.62±42.07
	GG	1.45±0.20	1141.50±52.90	507.12±36.47

All the data in the table are least-square means ± S.E. ^a,b^ Different superscripts within the same column demonstrate significant differences at *P* < 0.05.

**Table A4.** Association between different genotypes of detected SNPs in *GHR* 5’ UTR gene fragment and milk production-related traits.

SNP	Genotype	Average milk yield (L/day)	Average lactation length (days)	Average total lactation milk yield (L)
rs109014416	GG	6.01±0.65	272.36±29.60	1537.04±324.62
	GT	4.37±0.82	226.58±34.69	1519.23±380.39
	TT	5.46±0.37	280.35±15.39	1737.20±168.76
rs109231659	GG	5.52±0.37	271.68±16.41	1623.42±175.35
	GT	6.09±0.45	278.38±30.58	1761.47±326.71
	TT	8.12±0.84	242.96±40.87	1894.00±436.73
rs134115846	AA	6.20±0.55	284.51±23.14	1841.22±244.54
	AG	5.61±0.59	284.34±34.00	1837.76±268.17
	GG	5.17±0.52	263.79±21.55	1672.57±227.75

All the data in the least-square means ± S.E.
